# Eravacycline monotherapy and combination therapy against KPC-2- and NDM-1-co- producing *Klebsiella pneumoniae*: *in vitro* and *in vivo* activity analysis

**DOI:** 10.3389/fmicb.2026.1802577

**Published:** 2026-03-25

**Authors:** Xiaojie Zhao, Jingwei Wang, Xinyi Deng, Mengjiao Shi, Shuang Song, Shulong Zhao, Jingfang Sun, Youzhen Ma, Wenwen Zhu, Fei Jiang, Jing Tong, Haiquan Kang

**Affiliations:** 1 Department of Laboratory Medicine, Affiliated Hospital of Xuzhou Medical University, Xuzhou, Jiangsu, China; 2 The Center for Clinical Research and Transformation of Pathogen Diagnosis, Xuzhou Medical University, Xuzhou, Jiangsu, China; 3 Department of Burn and Plastic Surgery, The Affiliated Hospital of Xuzhou Medical University, Xuzhou, Jiangsu, China; 4 School of Medical Technology, Xuzhou Medical University, Xuzhou, China; 5 School of Life Sciences, Xuzhou Medical University, Xuzhou, Jiangsu, China; 6 Xuzhou Center for Disease Control and Prevention, Xuzhou, Jiangsu, China; 7 Precision Medicine Center, Xuzhou Medical University, Xuzhou, Jiangsu, China

**Keywords:** combination therapy, eravacycline, *Klebsiella pneumoniae*, KPC-2, NDM-1, synergy

## Abstract

**Background:**

The emergence of carbapenem-resistant *Klebsiella pneumoniae* (CRKP) co-producing KPC-2 and NDM-1 (K2N1-CRKP) has intensified a major public health threat and severely limited therapeutic options. Eravacycline, a novel tetracycline, shows potent *in vitro* activity against carbapenem-resistant Enterobacteriaceae. However, its efficacy and synergistic potential against K2N1-CRKP remain unclear.

**Methods:**

We analyzed the molecular epidemiology of 42 K2N1-CRKP strains and evaluated their susceptibility to eravacycline. The *in vitro* and *in vivo* (using a *Galleria mellonella* model) synergistic effects of eravacycline combined with other antibiotics were determined.

**Results:**

All 42 strains belonged to ST11. Among them, 92.8% (39/42) were susceptible to eravacycline (MIC50/90: 1 μg/mL; range: 0.5–2 μg/mL). Two strains (KP17, KP18) were resistant to both eravacycline and polymyxin B. The eravacycline-polymyxin B combination showed synergistic activity against 9.5% (4/42) of isolates, including the two dual-resistant strains. No interaction was observed for eravacycline combined with ceftazidime-avibactam, meropenem, or amikacin. Time-kill assays confirmed the rapid, sustained bactericidal activity of the eravacycline-polymyxin B combination against K2N1-CRKP. In the *G. mellonella* infection model, this combination provided a significantly superior survival benefit compared to monotherapy.

**Conclusion:**

Eravacycline demonstrates potent *in vitro* activity against ST11 K2N1-CRKP. While combinations with ceftazidime-avibactam, amikacin, or meropenem showed only additive effects, eravacycline plus polymyxin B exhibited strong synergistic and bactericidal activity both *in vitro* and *in vivo*, even against strains resistant to either agent alone. This regimen represents a promising evidence-based therapeutic option for these formidable infections.

## 1 Introduction

In recent years, the global prevalence of carbapenem-resistant *Klebsiella pneumoniae* (CRKP) has become a significant public health concern. The all-cause mortality rates of patients with CRKP infections in China, the United States, and South America are 24.2%, 23%, and 28%, respectively (Lou et al., 2022; Wang et al., 2022). Few antimicrobial agents have shown promising clinical efficacy for treating CRKP infections (Tian et al., 2021; Zheng et al., 2022). Ceftazidime-avibactam (CZA), approved for domestic marketing in 2019, has become the primary strategy and last-line drug for treating CRKP infections (Zheng et al., 2022; Cui et al., 2020). However, it only inhibits class A, class C (AmpC), and class D (e.g., OXA-48) β-lactamases, with no inhibitory effect on class B metalloenzymes represented by NDM-type enzymes (Cui et al., 2020; Tamma et al., 2022; Huang et al., 2023). Of particular concern is the emergence of *K. pneumoniae* co-producing KPC-2 (serine carbapenemase) and NDM-1 (metallo-β-lactamase) (K2N1-CRKP), which renders CZA ineffective against this type of CRKP. Furthermore, the emergence of K2N1-CRKP has increased the clinical challenges in treating carbapenem-resistant Enterobacteriaceae, including CRKP, and the urgency to develop new drugs.

Eravacycline, a novel, fully synthetic tetracycline antibiotic, exerts broad-spectrum antimicrobial activity by inhibiting the bacterial 30S ribosomal subunit and shows significant *in vitro* activity against multidrug-resistant Gram-negative bacteria. Its MIC_50_ against CRKP is 0.5 μg/mL, significantly lower than that of tigecycline (Zou et al., 2023). Eravacycline is unaffected by carbapenemases and can be clinically used to treat CRKP. However, studies on the evolution of eravacycline resistance during monotherapy (e.g., upregulation of the AcrAB-TolC efflux pump mediated by Lon protease gene mutations) suggest exploring combination therapeutic strategies to curb the development of resistance (Xu et al., 2022). Therefore, studying the synergistic effects of eravacycline and other antimicrobial agents has become an important research focus. Studies have evaluated the *in vitro* activity of eravacycline against CRKP; however, these studies only focused on *K. pneumoniae* producing a single carbapenemase (Wu et al., 2024). Although the emergence of K2N1-CRKP has been reported worldwide (Huang et al., 2023; Boralli et al., 2023), the monotherapy effect of eravacycline on K2N1-CRKP producing dual enzymes and its synergistic effect with other drugs have not been reported.

This study aimed to analyze the molecular epidemiology and evolutionary pathways of 42 strains of K2N1-CRKP and determine the susceptibility of these strains to eravacycline, as well as the *in vitro* synergistic effect of eravacycline-based combination on these strains. In addition, a *Galleria mellonella* infection model was used to provide *in vivo* combined bactericidal effects to provide experimental evidence for the optimization of clinical treatment regimens.

## 2 Materials and methods

### 2.1 Isolation and identification of bacteria

A total of 42 non-repetitive CRKP isolates that were first clinically isolated at the Affiliated Hospital of Xuzhou Medical University between January 2021 and December 2023 were screened. According to the Clinical and Laboratory Standards Institute guidelines breakpoints, CRKP was defined as clinical strains resistant to carbapenems (including imipenem and meropenem).

All strains were identified using a MALDI-TOF MS mass spectrometer (Chongqing Zhongyuan Huiji Biotechnology Co., Ltd.). Strain confirmation and antimicrobial susceptibility testing were performed using a VITEK-2 Compact automatic microbial identification and susceptibility analyzer (BioMérieux, France). The disk diffusion method was used for CZA: a bacterial suspension with a turbidity of 0.5–0.63 McFarland units was uniformly spread on a Mueller-Hinton agar (MHA) plate, cultured at 37 °C for 16–20 h, and the size of the minimum inhibitory zone was measured. Interpretation was performed according to CLSI criteria, isolates with a zone diameter of ≥21 mm was considered susceptible, and a diameter ≤20 mm was considered resistant. The quality control strain used was *K. pneumoniae* ATCC700603.

This study was approved by the Ethics Committee of the Affiliated Hospital of Xuzhou Medical University (approval number: XYFY2024-KL143).

### 2.2 DNA extraction and sequencing result analysis

Genomic DNA was extracted from the 42 CRKP strains according to the instructions provided by the Bacterial Genomic DNA Rapid Extraction Kit (Sangon Biotech). The extracted DNA was sent to Beijing Novogene Technology Co., Ltd., for bacterial genome sequencing using the Illumina Xplus platform, and the results were assembled using Unicycler v0.4.7. MLST was performed using the website http://bacdb.cn/BacWGSTdb/Tools.php. Genomic drug resistance genes were analyzed using the website http://proksee.ca. After obtaining the next-generation sequencing (NGS) results, two representative isolates (KP17, KP23) were selected for full-length whole-genome sequencing.

### 2.3 Antimicrobial susceptibility testing

#### 2.3.1 Determination of single-drug MIC values using the 96-well plate micro-broth dilution method

The stock solutions of the four selected antibiotics were prepared; the initial drug concentration was set, and serial 2-fold dilutions were performed from high to low concentrations to generate 10 concentration gradients in 96-well plates, using cation-adjusted Mueller-Hinton broth (CAMHB) as the test medium. This was followed by inoculation with bacterial suspensions adjusted to a final concentration of 5 × 10^5^ CFU/mL. The plate was placed in a constant temperature incubator at 35 ± 2 °C for overnight culture.

After 16–20 h of culture, the results were observed, and the MIC value when the drug was used alone was recorded (the MIC value of the strain for the single drug was determined). Susceptibility to eravacycline was based on the breakpoint standards of the Expert Committee for the Research and Standardization of Clinical Antimicrobial Susceptibility Breakpoints of the National Health Commission of China; and susceptibility to other drugs was based on the breakpoint standards of the Clinical and Laboratory Standards Institute.

#### 2.3.2 Antibiotics and drug combinations

Eravacycline, polymyxin B, amikacin, ceftazidime-avibactam (CZA), and meropenem were commercially sourced from Macklin Inc. The monotherapy solutions of each drug and the combination therapy solutions (eravacycline paired with each of the other drugs individually) were prepared per the manufacturer’s operating protocols in cation-adjusted Mueller-Hinton broth (CAMHB). The required working concentrations were obtained via the serial 2-fold concentration gradient dilution method.

#### 2.3.3 Determination of synergistic effects

The checkerboard broth microdilution assay was employed to evaluate the synergistic antimicrobial effects of eravacycline in combination with polymyxin B, CZA, amikacin, and meropenem. All assays were performed in cation-adjusted Mueller-Hinton broth (CAMHB). Different eravacycline and polymyxin B concentrations were mixed and added to 96-well plates. The maximum concentrations of eravacycline and polymyxin B were 2 and 8 mg/L, respectively. After 16–20 h incubation, bacterial growth was recorded, and the FICI was calculated.

To ensure the reliability of the experimental data, each dilution was tested in triplicate. The synergistic effects were determined by calculating the FICI of the combination as follows: [(MIC of drug A tested in combination)/(MIC of drug A tested alone)] + [(MIC of drug B tested in combination)/(MIC of drug B tested alone)]. Synergy was defined as a FICI ≤ 0.5; no interaction was described as a FICI > 0.5 and ≤4; and antagonism was defined as a FICI of >4 (Fatsis-Kavalopoulos et al., 2024). *Escherichia coli* ATCC 25922 was used as the quality control strain.

#### 2.3.4 Time-kill curve analysis

The efficacy of each drug alone and in combination against *K. pneumoniae* was assessed using static time–kill assays. Four *K. pneumoniae* isolates (KP-4, KP-7, KP-8, and KP-23) that met the following criterion were selected: the combination of eravacycline and polymyxin B demonstrated synergy against these isolates. For each isolate, four treatment regimens were tested: placebo control; The concentrations of 1/2 MIC eravacycline and 1/2 MIC polymyxin B were selected for the time-kill kinetic assay as described previously (White et al., 1996); and the combination of eravacycline and polymyxin B at concentrations shown to be effective in the checkerboard assays. Representative isolates were inoculated into 25 mL of fresh CAMHB at an initial concentration of 5 × 10^∧^5 CFU/mL. Samples were collected at 0, 3, 6, 12, 24, and 48 h to evaluate the effects of single and combined drugs on the bacteria count. Synergistic activity was identified by a reduction of no less than 2 log_10_ CFU/mL in the combination group at 24 h when compared to the most potent single antibiotic (Doern, 2014), as well as *a* ≥ 2 log_10_ decrease relative to the bacterial count at 0 h (White et al., 1996).

#### 2.3.5 Galleria mellonella

Infection model *G. mellonella* larvae (Tianjin Huiyude Biotechnology Co., Ltd.) were used for *in vivo* experiments. First, the toxicity of eravacycline and polymyxin B, their combination in *G. mellonella* larvae, were evaluated as previously described (Betts et al., 2017). The criterion for no observable toxicity was defined as follows: within 96 h after drug administration, larval survival showed no significant difference compared with the PBS-injected control group (*P* > 0.05). After verifying that these two drugs had no significant toxicity to *G. mellonella* larvae, we assessed the *in vivo* therapeutic potential of the eravacycline-polymyxin B combination therapy.

Infection: According to a previous study with minor modifications (Zhang et al., 2021), in the preliminary experiment, *G. mellonella* were injected with bacterial suspensions at concentrations of 1.5 × 10^6^, 1.5 × 10^7^, and 1.5 × 10^8^ CFU/ml. The concentration that resulted in 80% mortality in *G. mellonella* within 24–48 h was chosen as the inoculum concentration. *G. mellonella* larvae were infected by injecting 10 μL of a suspension containing KP-4 (1.5 × 10^∧^8 CFU/ml), KP-17 (1.5 × 10^∧^8 CFU/ml), KP-18 (1.5 × 10^∧^8 CFU/ml), or KP-23 (1.5 × 10^∧^8 CFU/ml) into the left foreleg.

Treatment: Thirty minutes after infection, the larvae were randomly divided into four groups (10 larvae per group); the phosphate-buffered saline (PBS)-treated group was the infected untreated control (i.e., bacteria + PBS injection) in our study, eravacycline, polymyxin B, or the eravacycline-polymyxin B combination was injected into the right foreleg. The target hemolymph concentrations were set at 1–2 × MIC. The dose (mg/kg) was calculated as follows: Dose (mg/kg) = N × MIC (μg/mL) × Vtotal (μL)/Larval weight (mg), where *N* represents the multiple of the MIC (1 × or 2 ×), Vtotal = 60 μL, which includes the estimated larval hemolymph volume (40 μL) and the total injection volume (20 μL), and the average larval weight was set at 250 mg (Tsai et al., 2016). All concentrations of the test drugs employed in this experiment exhibited no significant toxicity to *G. mellonella* larvae.

The larvae were cultured in a Petri dish at 37 °C, and the survival rates were recorded at 0, 12, 24, 48, 60, 72, 84, and 96 h. A larva was considered dead if it showed no response to external stimuli.

All experiments were performed in triplicate independently. The final results are presented as the mean survival rates from the combined experimental data.

#### 2.3.6 Statistical analysis

GraphPad Prism 8.0.2 was employed for all statistical analyses. Data from *in vitro* assays were generated from three separate experiments, and values are shown as mean ± SD. Survival rates in the *G. mellonella* model were compared using the log-rank test. A probability level of *P* < 0.05 was defined as statistically significant.

## 3 Results

### 3.1 Departmental distribution of K2N1-CRKP

A total of 42 CZA-resistant *K. pneumoniae* strains were clinically isolated. Next - generation sequencingwas used to screen for drug resistance genes, and all 42 strains harbored both *bla*_KPC–2_ and *bla*_*NDM*_.

### 3.2 Phenotypic and microbiological characteristics of K2N1-CRKP

#### 3.2.1 Molecular epidemiological characteristics and whole-genome sequencing result

**FIGURE 1:**
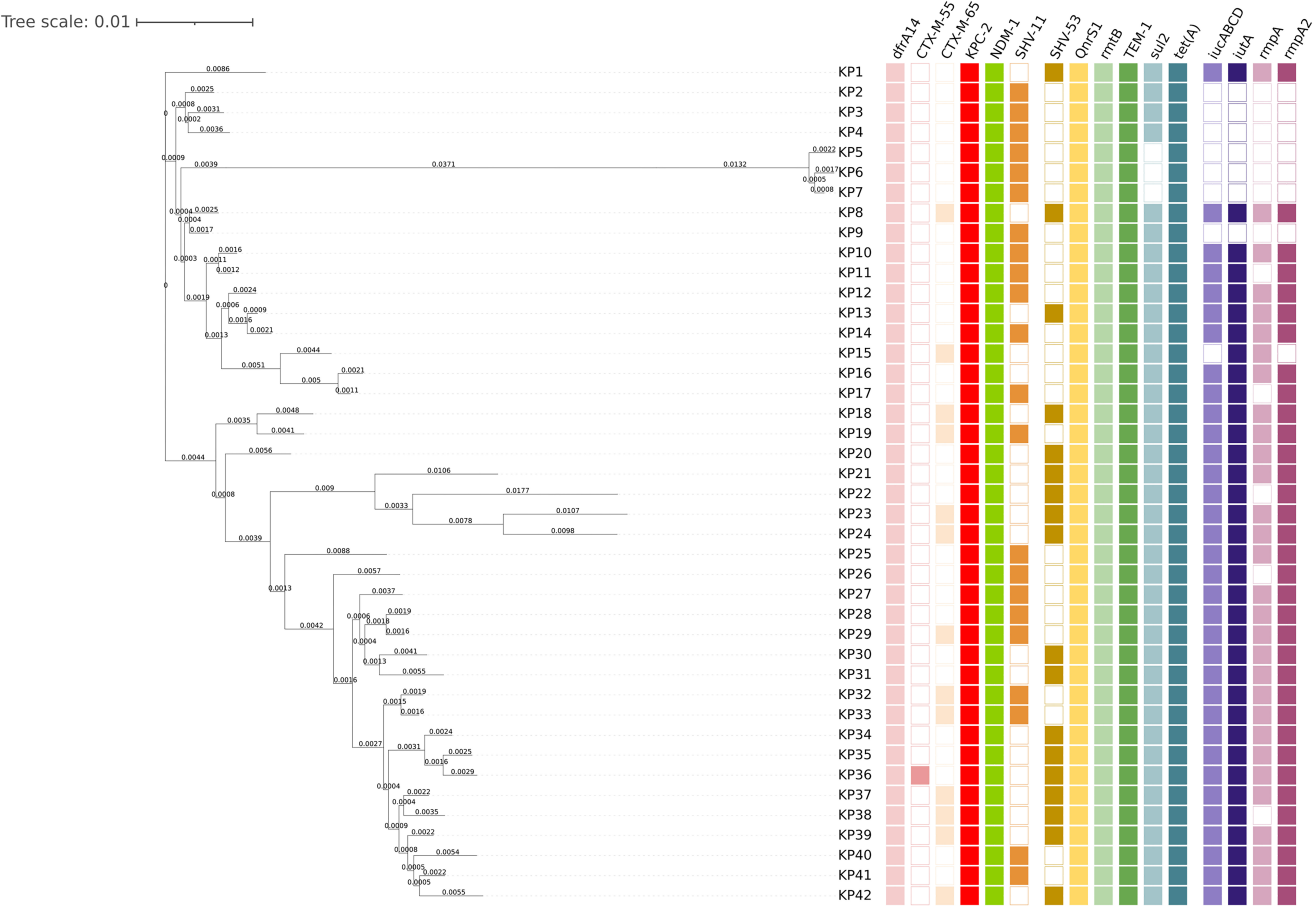
Phylogenetic analyses of 42 *Klebsiella pneumoniae* strains co-carrying *blaKPC* and *blaNDM* genes. Cells of different colors represent wards carrying various antimicrobial resistance genes or virulence genes, while blank cells indicate the absence of the corresponding genes.

Next-generation sequencing was performed on all the isolates to comprehensively elucidate the molecular epidemiological characteristics of the 42 strains (Figure 1). The results showed that all strains (100%, 42/42) harbored the *bla*_KPC–2_ and *bla*_*NDM*_ genes; 57.1% (24/42) of the strains harbored the *bla*_SHV–11_ gene; 42.9% (18/42) harbored the *bla*_SHV–53_ gene; 33.3% (14/42) harbored the *bla*_CTX–*M–65*_ gene; one strain harbored the *bla*_CTX–M–55_ gene; and one strain harbored the *arr-2* gene. Notably, all *K. pneumoniae* strains simultaneously harbored the *bla*_TEM–1_, *tet(A)*, *dfrA14*, or *Qnrs1* gene (see Supplementary Table 1). Multilocus sequence typing (MLST) showed that the housekeeping genes of all strains were *gapA_3*, *infB_3,mdh_1*, *pgi_1*, *phoE_1*, *rpoB_1*, and *tonB_4*, which belong to the ST11 type, the most common type in China. The complete genome sequences of KP17 and KP23 have been deposited in the NCBI GenBank database under the BioProject accessions PRJNA1292292 and PRJNA1292749, respectively.

#### 3.2.2 Antimicrobial susceptibility results of 42 strains of K2N1-CRKP

The broth microdilution method determined the minimum inhibitory concentrations (MICs) of eravacycline, polymyxin B, CZA, amikacin, and meropenem for all tested strains. As shown in Table 1, the MIC range of eravacycline was 0.5–2 μg/mL, with an MIC_50_ of 1 μg/mL and an MIC_90_ of 1 μg/mL; 7.1% (3/42) of the strains were resistant to eravacycline. The MIC range of polymyxin B for all tested strains was 0.5–8 μg/mL, with an MIC_50_ of 1 μg/mL and an MIC_90_ of 1 μg/mL; 4.8% (2/42) of the strains were resistant to polymyxin B. Among them, KP17 and KP18 were resistant to both polymyxin and eravacycline. All strains were resistant to amikacin, with an MIC range of 32–512 μg/mL, an MIC_50_ of 256 μg/mL, and an MIC_90_ of 512 μg/mL. Furthermore, all strains were resistant to CZA, with an MIC range of 128–512 μg/mL, an MIC_50_ of 256 μg/mL, and an MIC_90_ of 512 μg/mL. Additionally, all strains were resistant to meropenem, with an MIC range of 128–512 μg/mL, an MIC_50_ of 512 μg/mL, and an MIC_90_ of 512 μg/mL. Detailed results are listed in Supplementary Table 2.

**TABLE 1:** The minimum inhibitory concentration (MIC) values and resistance rates of eravacycline and other drugs.

Species (no.)	Drug	MIC (μ g/mL)-MIC_50_	MIC (μ g/mL)-MIC_90_	MIC (μ g/mL)-range	R%
K2N1-CRKP (*n* = 42)	Eravacycline	1	1	0.5–2	7.1
	Polymyxin B	1	1	0.5–8	4.8
	Amikacin	256	256	32–256	100
	CZA	256	256	32–256	100
	Meropenem	512	512	128–512	100

### 3.3 Comparison of MIC values between single and combined drug applications

**FIGURE 2:**
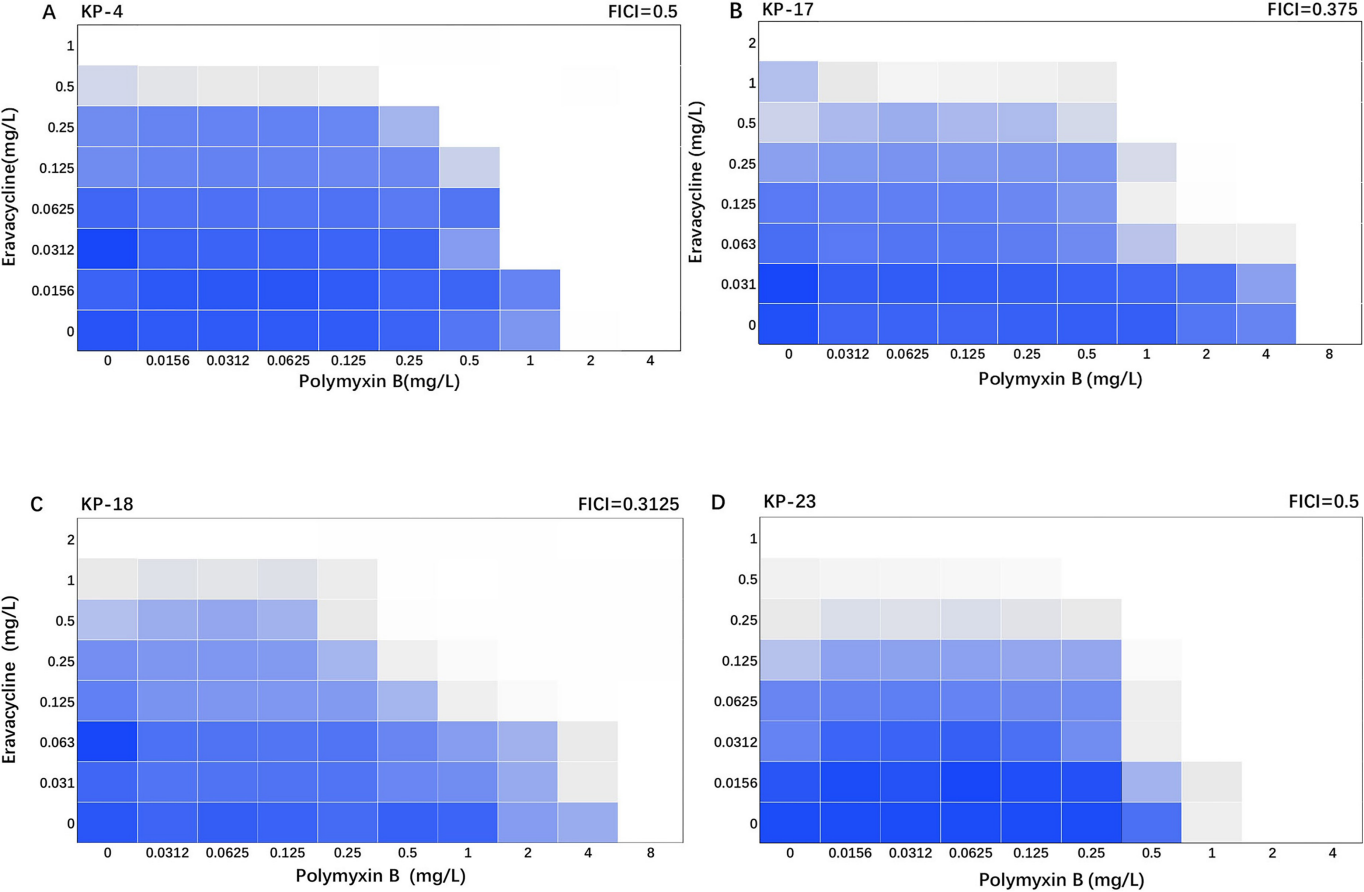
**(A–D)** Shows the synergistic activity of eravacycline and polymyxin B against *Klebsiella pneumoniae* KP-4, KP-17, KP-18, and KP-23, respectively. The average OD600 values were recorded after 18 h of incubation. The dark blue area represents a higher bacterial density.

Table 2 shows that no antagonistic effects were observed for any tested strains. The eravacycline-polymyxin B combination showed significant synergistic effects (fractional inhibitory concentration index [FICI] ≤ 0.5) on four (9.52%) isolates (KP4, KP17, KP18, and KP23), the FICI values were 0.5, 0.375, 0.313, and 0.5, respectively (Figures 2A–D). Among the four isolates, the polymyxin B-resistant strains KP-18 and KP-17 had a polymyxin B monotherapy MIC of 8 μg/mL. After combination with eravacycline, the polymyxin B MICs decreased to 1 and 0.5 μg/mL (Table 3), respectively, restoring susceptibility to polymyxin B. No interaction was observed for eravacycline combined with ceftazidime-avibactam, meropenem, or amikacin. Detailed results are listed in Supplementary Table 2.

**TABLE 2:** Results of chequerboard analysis for eravacycline-based combinations in K2N1-carbapenem-resistant *Klebsiella pneumoniae* (K2N1-CRKP) isolates.

Species (no.)	Drug combination	Synergistic-no. (%)	No interaction-no. (%)	Antagonism-no. (%)
K2N1-CRKP (*n* = 42)	Eravacycline + polymyxin B	4 (9.5)	38 (90.5)	0 (0)
	Eravacycline + amikacin	0 (0)	42 (100)	0 (0)
	Eravacycline + CZA	0 (0)	42 (100)	0 (0)
	Eravacycline + meropenem	0 (0)	42 (100)	0 (0)

**TABLE 3:** Fractional inhibitory concentration index (FICI) values for eravacycline/polymyxin B combinations against K2N1-carbapenem-resistant *Klebsiella pneumoniae* (K2N1-CRKP).

strains	Monotherapy (μg/ml)		Combination (μg/ml)		FICI	Interpretition
	Eravacycline	Polymyxin B	Eravacycline	Polymyxin B		
KP4	1	2	0.25	0.5	0.5	Synergistic
KP17	2	8	0.5	1	0.375	Synergistic
KP18	2	8	0.5	0.5	0.3125	Synergistic
KP23	1	2	0.25	0.5	0.5	Synergistic

### 3.4 Time-kill curves

**FIGURE 3:**
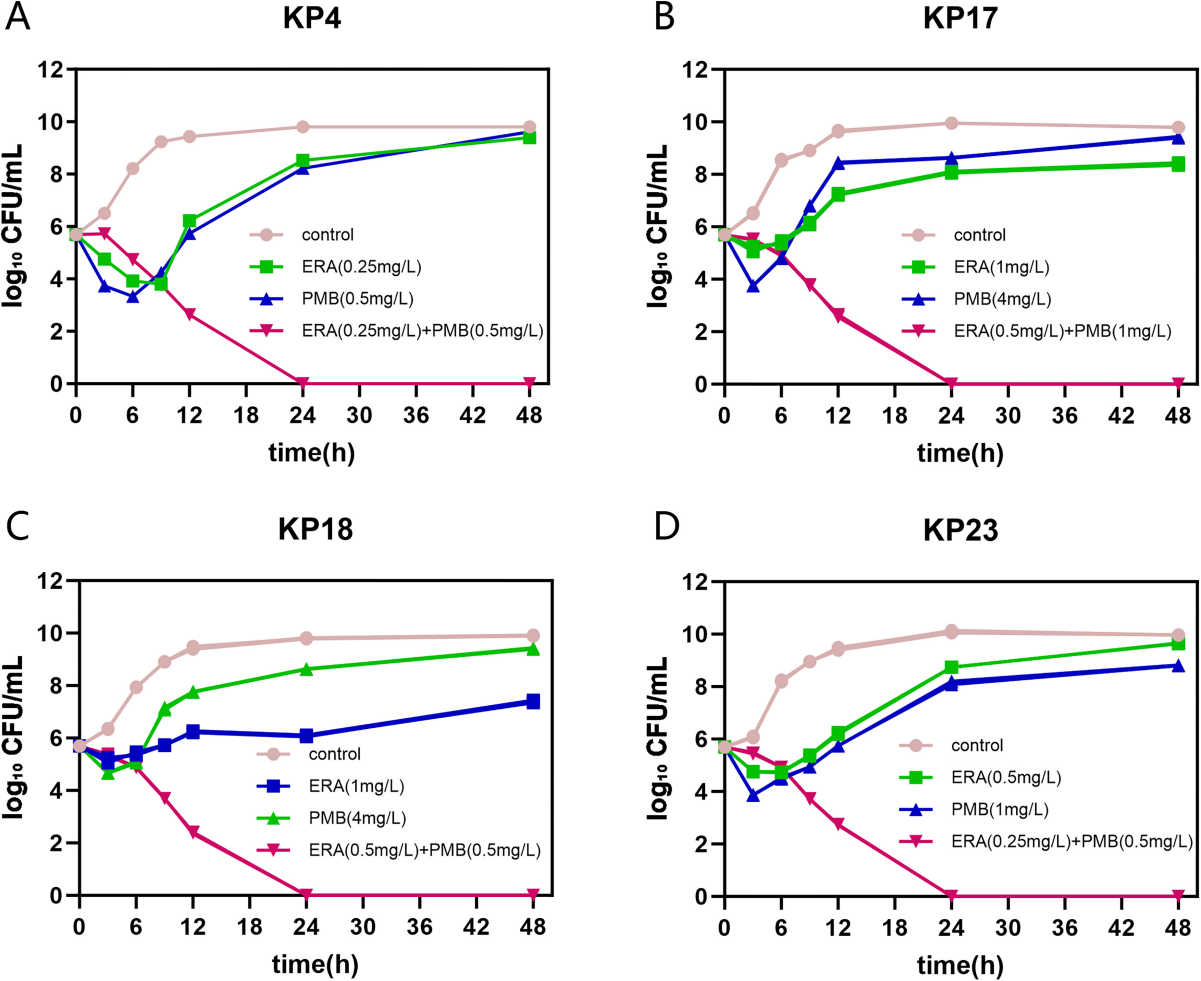
Time-kill curves showing the antimicrobial effects of the combination of eravacycline and polymyxin B against *Klebsiella pneumoniae* KP-4 **(A)**, KP-17 **(B)**, KP-18 **(C)**, and KP-23 **(D)**. Synergy was defined as a ≥ 2 log_10_ reduction in bacterial count by the combination compared with the most effective single agent.

Time-kill curves were used to evaluate the bactericidal effects of the combination of eravacycline and polymyxin B against four isolates, namely KP-4, KP-17, KP-18, and KP-23 (Figures 3A–D). The experimental results showed that monotherapy with eravacycline at 1/2 MIC and polymyxin B monotherapy at 1/2 MIC resulted in an initial decrease in bacterial counts; however, bacterial regrowth was observed in all four isolates within 6–24 h. In contrast, in the combination therapy, the eravacycline-polymyxin B combination exhibited rapid bactericidal activity, and no bacterial regrowth was observed within 24 h (below the detection limit, <10 CFU/mL); this inhibition lasted up to 48 h. Compared with the eravacycline or polymyxin B monotherapy groups, the bactericidal activity of the eravacycline-polymyxin B combination was significantly enhanced, and the colony counts of all four isolates were reduced by >2 log_10_ CFU/mL at 24 h compared with the monotherapy groups.

### 3.5 *Galleria mellonella* infection model

**FIGURE 4:**
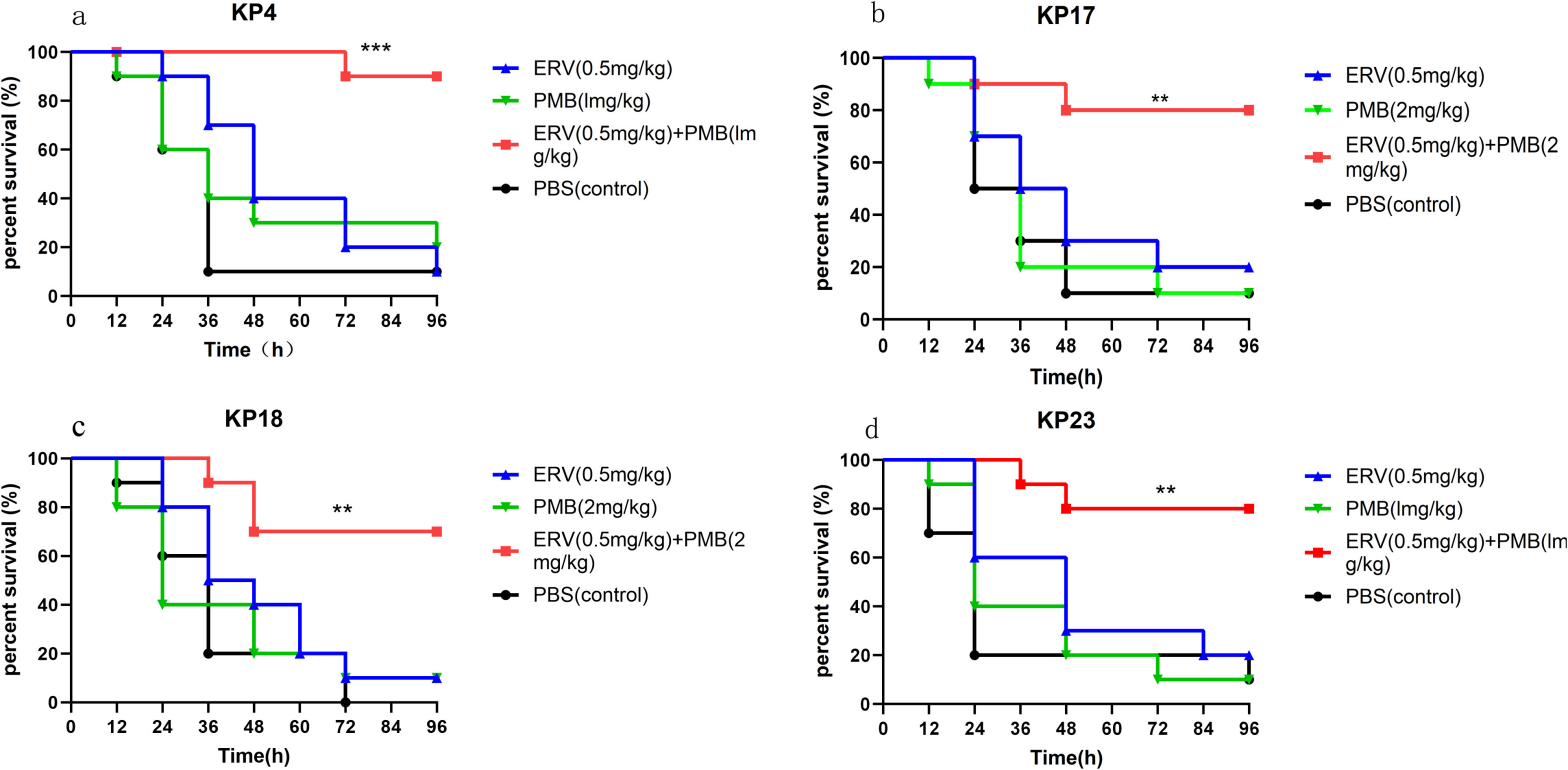
*S*urvival curves showing the synergistic effect of eravacycline and polymyxin B in *Galleria mellonella* larvae infected with *Klebsiella pneumoniae* KP-4 **(a)**, KP-17 **(b)**, KP-18 **(c)**, and KP-23 **(d)** (** indicates *P* < 0.01; *** indicates *P* < 0.001).

A *G. mellonella* larval infection model was established to evaluate the *in vivo* therapeutic potential of the combination of eravacycline and polymyxin B against *K. pneumoniae* (KP-4, KP-17, KP-18, and KP-23) (Figure 4). The experimental results showed that, compared with the use of either drug alone, the eravacycline-polymyxin B combination therapy significantly increased the larval survival rate (*P* < 0.01). There was no significant difference in the larval survival rate for each strain between the monotherapy and control groups (*P* > 0.1). The specific results are discussed below.

Larvae infected with *K. pneumoniae* KP-4 (Figure 4a): The combination therapy (0.5 mg/kg eravacycline + 1 mg/kg polymyxin B) significantly increased the survival rate compared to eravacycline monotherapy (90% vs. 20%, *P* < 0.001) and polymyxin B monotherapy (90% vs. 30%, *P* < 0.001). Larvae infected with *K. pneumoniae* KP-17 (Figure 4b): Combination therapy (1 mg/kg eravacycline + 2 mg/kg polymyxin B) significantly increased the survival rate compared to eravacycline monotherapy (80% vs. 20%, *P* = 0.009) and polymyxin B monotherapy (80% vs. 10%, *P* = 0.002). Larvae infected with *K. pneumoniae* KP-18 (Figure 4c): The survival rates after eravacycline monotherapy and polymyxin B monotherapy were 20% and 10%, respectively, whereas combination therapy (1 mg/kg eravacycline + 1 mg/kg polymyxin B) resulted in a survival rate of 70% (*P* = 0.003 and *P* = 0.007, respectively). Larvae infected with *K. pneumoniae* KP-23 (Figure 4d): The survival rate of larvae receiving the combination therapy was higher than that of larvae receiving eravacycline monotherapy (80% vs. 20%, *P* = 0.008) or polymyxin B monotherapy (80% vs. 10%, *P* = 0.001).

These results indicated that eravacycline-polymyxin B combination therapy significantly increased the survival rate of *G. mellonella* larvae infected with multidrug-resistant bacteria in *in vivo* experiments, demonstrating its effectiveness as a potential therapeutic strategy.

## 4 Discussion

The emergence of *K. pneumoniae* producing KPC-2 and NDM-1 carbapenemases poses a severe threat to global public health. Owing to the almost pan-drug resistance of these strains, they are closely associated with high mortality rates in clinical settings (Lou et al., 2022; Wang et al., 2022). The ratio of KN-CRKP globally in *K. pneumoniae* significantly increased from 0.03% to 3.10% during 2014–2023, especially from 2017 to 2023 (Zhang et al., 2025). This study is the first to conduct a comprehensive and in-depth analysis of 42 clinically isolated strains harboring the *bla*_KPC–2_ and *bla*_NDM–1_ genes, with all 42 isolated strains belonging to the ST11 type. Sequence type 258 (ST258) is an internationally high-risk epidemic CRKP lineage relatively common in Europe and the United States (Wyres et al., 2020). Meanwhile, ST11 is the most common CRKP clone in China, and it is part of the clonal complex 258 (CC258) and a single-locus variant of ST258 (Liao et al., 2020). This difference reflects the regional specificity of the transmission and evolution of bacterial resistance in different regions.

The emergence of K2N1-CRKP may be due to the acquisition of a highly transferable *bla*_NDM–1_ plasmid by *bla*_KPC–2_ CRKP (Gao et al., 2020). In 2019, Huang et al., reported a case in which the *bla*_KPC–2_ CRKP strain acquired a *bla*_NDM–5_ plasmid with a low fitness cost and transformed it into a dual-enzyme phenotype under CZA pressure (Huang et al., 2021). Wang et al. (2024) reported that *bla*_KPC–2_-carrying CRKP strains acquired a plasmid carrying the *bla*_NDM–1_ gene following treatment with trimethoprim-sulfamethoxazole (SXT), thereby evolving into KPC-2- and NDM-1-co-producing CRKP. Notably, this *bla*_NDM–1_-harboring plasmid facilitated the transfer of the previously non-conjugative *bla*_KPC–2_ plasmid. The frequent co-localization of *bla*_KPC–2_-harboring plasmids with hypervirulence determinants suggests that dual-carbapenemase-producing strains can concurrently acquire enhanced virulence and resistance phenotypes (Cai et al., 2026). This convergence underscores the clinical urgency of developing effective therapeutic strategies specifically targeting K2N1-CRKP. Molecular epidemiological analysis revealed that the IncFII/IncR plasmid is the primary carrier of *bla*_KPC–2_, while the IncX3, IncN, and IncFIB/IncFII plasmids are the predominant carriers of *bla*_NDM–1_ (Cai et al., 2026). In this study, the *bla*_KPC–2_ gene harbored by strain KP17 was localized to an IncFII/IncR-type plasmid of 133,775 bp in length, which represents the primary vector for *bla*_KPC–2_ to date. In contrast, the *bla*_NDM–1_ gene was found on an untypable plasmid with a size of 102,184 bp. We speculate that K2N1-CRKP may have been generated by ST11-type *bla*_KPC–2_ CRKP acquiring a plasmid carrying *bla*_NDM–1_ exogenously under the selection pressure of CZA. K2N1-CRKP resulted from the combined action of antibiotic selection pressure and the horizontal transfer of bacterial resistance genes. Its high resistance to CZA poses an urgent challenge to clinical treatment, necessitating the development of new antimicrobial agents.

As a novel tetracycline antibiotic, eravacycline exhibits good antimicrobial activity against enzyme-producing strains. Moreover, whether CRKP produces KPC or NDM enzymes, eravacycline exhibits good *in vitro* antimicrobial effects (Zou et al., 2023). In this study, 95.2% (39/42) of the 42 strains of K2N1-CRKP were susceptible to eravacycline (MIC ≤ 1), indicating that eravacycline has a good *in vitro* antimicrobial effect against K2N1-CRKP. Its MIC_50/90_ values were 1/1 μg/mL, and the MIC_50_ was higher than the MIC_50_ of 0.38 μg/mL reported in previous study (Wang et al., 2024). This difference may reflect significant differences in the resistance mechanisms between the different regions. With the growing clinical use of eravacycline, the emergence and transmission of its resistance have garnered widespread attention in academic and clinical circles. To extend eravacycline’s clinical utility and curb the prevalence and spread of resistance, clinical laboratories should perform antimicrobial susceptibility testing and resistance gene screening prior to its prescription to support precise medication.

Carbapenem-resistant *Klebsiella pneumoniae* infections with *bla*_KPC–2_ and *bla*_NDM–1_ have occasionally been reported globally (Boralli et al., 2023; Rong et al., 2023). Gao et al. (2020) screened 2057 non-repetitive clinically isolated CRKP strains collected from 65 hospitals in 25 provinces and cities in China and identified seven K2N1-CRKP strains. These seven K2N1-CRKP strains were resistant to various antimicrobial agents but were fully susceptible to polymyxin and tigecycline. However, in this study, two K2N1-CRKP strains were resistant to polymyxin B, and three were resistant to eravacycline. Notably, two of these strains were resistant to both eravacycline and polymyxin B. Polymyxins (including colistin and polymyxin B) are the last treatment options for carbapenem-resistant Enterobacterales infections (Macesic et al., 2020); therefore, polymyxin B resistance in 4.8% (2/42) of the isolated strains, as reported in the present study, is notable. Therefore, the present study identified that 4.8% (2/42) of the isolated strains exhibited concurrent resistance to polymyxin B, eravacycline, and ceftazidime-avibactam, a finding that carries significant clinical implications. Given the prolonged development cycles and substantial challenges associated with the approval and commercialization of novel antimicrobial agents, developing effective therapeutic strategies based on currently available antimicrobials to manage infections caused by such extensively drug-resistant (XDR) bacteria has emerged as a critical unmet need in contemporary clinical infectious diseases.

The 42 K2N1-CRKP strains in this study were 100% resistant to CZA (42/42), whereas the rate of resistance to eravacycline was only 7.1% (3/42). This suggests that eravacycline is an effective drug to treat K2N1-CRKP infections. With the use of eravacycline, there have been an increasing number of reports of related resistance. Therefore, extending the service life of eravacyclines through effective combination therapies is particularly important. We used the microdilution checkerboard method to detect the combined effects of eravacycline with polymyxin B, CZA, amikacin, and meropenem. The results showed that the combination of eravacycline and polymyxin B exhibited a synergistic relationship (FICI of 0.0375–0.50) against 9.52% (4/42) of the strains and no interaction (FICI of 0.75–4) on 38 strains. Brennan-Krohn and Kirby (2019) reported that the combination of polymyxin B and eravacycline showed a synergistic antimicrobial effect on one CRKP strain, AR-0636, carrying *bla*_NDM–1_. The MICs of eravacycline and polymyxin B for this strain were 4 and 16 μg/ml, respectively. When the two drugs were combined, eravacycline and polymyxin B concentrations decreased to 0.25 and 1 μg/ml, respectively.

Regarding KP17, when eravacycline and polymyxin B were used in combination, the respective MICs changed from the original 2 and 8 to 0.5 and 1 μg/ml; meanwhile, for KP18, the respective MICs changed from the original 2 and 8 to 0.5 and 0.5 μg/ml. This indicated that combining the two drugs produced a strong synergistic antimicrobial effect, significantly reducing the dosage required for each drug to reach an effective antimicrobial concentration. Eravacycline combined with CZA showed no interaction on 42 strains. Although CZA has a strong inhibitory effect on KPC-2, it has no inhibitory effect on NDM, which may explain its low synergistic effect when combined with eravacycline (Li et al., 2022).

Li et al. (2022) reported synergistic effects in 25% and 5% of bla_KPC–2_-carrying CRKP strains when tested with eravacycline-amikacin and eravacycline-imipenem, respectively. However, *in vitro* antimicrobial susceptibility testing of our 42 K2N1-CRKP strains revealed no interaction effects with the two combination regimens (eravacycline/amikacin and eravacycline/imipenem). The absence of synergy in our study is likely attributable to the complex genetic background of the strains. All 42 CRKP strains co-harbored the *bla*_KPC–2_ and *bla*_NDM–1_ carbapenemase genes, which may have attenuated the synergistic potential of the drug combinations through multiple concurrent resistance mechanisms. Furthermore, the high minimum inhibitory concentrations (MICs) of amikacin (128–512 μg/mL) and imipenem (256–512 μg/mL) indicate strong intrinsic resistance to these agents, further compromising the potential for synergistic antibacterial activity with eravacycline. In addition, for the 42 K2N1-CRKP strains, both eravacycline and polymyxin B alone had high susceptibility rates, and synergistic effects were observed in two strains (KP-17 and KP-18) that were resistant to both polymyxin B and eravacycline. This finding indicates that the combination of eravacycline and polymyxin represents a viable strategy for reversing or overcoming bacterial resistance to eravacycline, demonstrating potential clinical relevance. Notably, mechanistic analysis revealed that 41 out of the 42 strains (97.62%) harbored the 16S rRNA methyltransferase gene rmtB. This gene encodes an enzyme that methylates the aminoglycoside-binding site on the 16S rRNA, leading to high-level cross-resistance to all clinically relevant aminoglycosides, including amikacin. This finding provides a molecular explanation for the observed high amikacin MICs and, consequently, the failure of the eravacycline-amikacin combination.

The efficacy of eravacycline-polymyxin B combination therapy was verified through *in vitro* time-kill experiments, which dynamically monitored the bactericidal activity of the combination therapy over time. The combination of 0.5 μg/mL eravacycline and 1 μg/mL polymyxin B effectively inhibits the growth of eravacycline-polymyxin B-resistant strains KP17 and KP18; additionally, the resistant strains restored susceptibility to eravacycline and polymyxin B. This indicates that eravacycline can enhance the antimicrobial activity of polymyxin B at low concentrations, thereby effectively overcoming drug resistance. *In vitro* time-kill experiments showed that polymyxin B monotherapy was ineffective after 6 h. Meanwhile, the addition of eravacycline continuously reduced the bacterial count, and complete inhibition of bacterial growth was achieved within 24 h, with this effect lasting up to 48 h. These results indicate that the combination of the two drugs enhances the inhibitory effect on bacteria, restores the susceptibility of resistant bacteria to eravacycline and polymyxin B, and exerts advantages over monotherapy.

We infected *G. mellonella* with KP4, KP17, KP18, and KP23 and administered monotherapy and combination therapy. The results showed that combination therapy significantly increased the larval survival rate from 10% during monotherapy to 70%–80%, indicating that the combination of eravacycline and polymyxin B significantly improved the therapeutic effect against K2N1-CRKP in the insect model. The eravacycline-polymyxin B combination demonstrates synergistic antibacterial activity in both *in vitro* and *in vivo* models. It is a valuable therapeutic option with particular clinical significance for regions like China, where the ST11 clone is prevalent. This is especially critical for managing strains that exhibit dual resistance to both agents. The clinical utility of polymyxin B is primarily limited by its dose-dependent nephrotoxicity, with the incidence of acute kidney injury (AKI) ranging from 34.8% to 42.9% due to mechanisms such as renal tubular accumulation and oxidative stress (Wang et al., 2022; Wen et al., 2025). The synergistic interaction observed between polymyxin B and eravacycline (FICI ≤ 0.5) offers a potential strategy to mitigate this toxicity by enabling a significant reduction in the dose of polymyxin B while preserving antibacterial efficacy. Further pharmacokinetic/pharmacodynamic (PK/PD) studies and well-designed clinical trials are warranted to validate whether this regimen can improve the therapeutic index of polymyxin B without compromising clinical outcomes.

This study systematically evaluated the *in vitro* antibacterial activity of eravacycline against ST11-type K2N1-CRKP. The results demonstrated potent bactericidal activity against these strains, suggesting its potential as a novel therapeutic candidate for infections caused by this dual-carbapenemase-positive pathogen. Both *in vitro* and *in vivo* experimental data confirmed that the combination of eravacycline and polymyxin B can effectively reverse eravacycline resistance in the strains, exhibiting a clear synergistic antibacterial effect. This finding challenges the traditional notion that “resistance equates to therapeutic ineffectiveness,” indicating that antimicrobial agents with established resistant phenotypes can regain their clinical application potential through rational combination strategies. Notably, in the study targeting ST11-type K2N1-CRKP, no significant synergistic effect was observed when eravacycline was combined with amikacin, meropenem, or ceftazidime-avibactam, which underscores the specificity and critical importance of selecting appropriate combination regimens. The clinical translation of this optimized combination strategy is expected to prolong the effective clinical lifespan of key antimicrobial agents and provide a novel therapeutic approach for the treatment of multidrug-resistant bacterial infections.

Firstly, although this study provides the first detailed insight into the activity of eravacycline-based combinations against ST11 K2N1-CRKP coproducing dual carbapenemases, the relatively modest sample size and single-center design may limit the statistical power and generalizability of the conclusions. Future multicenter studies with larger cohorts are warranted to validate these observations. Secondly, while our *in vitro* and *G. mellonella* models offer a robust foundation for evaluating synergistic effects, they inherently cannot fully recapitulate the complex pharmacokinetic and immunological environment of the human host. Moreover, the mechanisms underlying the observed synergy were not explored at the transcriptomic level, representing an important avenue for future mechanistic research. Thirdly, it is important to note that novel agents such as aztreonam-avibactam and cefiderocol were not included in this comparative analysis. While our study focused on evaluating synergies among widely accessible antibiotics—a question of high relevance in resource-limited settings where newer agents are not yet routinely available—we acknowledge that the absence of these emerging drugs limits the comprehensiveness of our study in the context of the current antimicrobial landscape. Future investigations should therefore incorporate these novel compounds to benchmark the relative efficacy of the combinations tested here. Despite these limitations, our findings provide a critical preclinical rationale for considering eravacycline-polymyxin B combinations as a therapeutic option against this challenging resistance phenotype, and highlight specific directions for subsequent clinical and mechanistic validation.

In conclusion, our study delineates the potent activity of eravacycline against the formidable ST11 K2N1-CRKP clone and identifies eravacycline-polymyxin B as a synergistic combination capable of rescuing efficacy even against isolates resistant to both agents. These findings challenge the notion of irreversible resistance and highlight the imperative for pathogen-tailored combination strategies. While further mechanistic and preclinical studies are warranted, the eravacycline-polymyxin B regimen emerges as a critically needed, evidence-based option for managing these life-threatening infections, especially in regions dominated by the ST11 clone.

## Data Availability

The original contributions presented in this study are included in this article/Supplementary material, further inquiries can be directed to the corresponding authors.

## Supplementary material

Supplementary Table 1 Next-generation sequencing results of 42 K2N1-carbapenem-resistant *Klebsiella pneumoniae* (K2N1-CRKP) isolates.

Supplementary Table 2 Minimum inhibitory concentration (MIC) values and fractional inhibitory concentration index (FICI) of eravacycline in combination with ceftazidime-avibactam, polymyxin B, meropenem, and amikacin in susceptibility testing.
